# Turnip Mosaic Virus Infection Differentially Modifies Cabbage Aphid Probing Behavior in Spring and Winter Oilseed Rape (*Brassica napus*)

**DOI:** 10.3390/insects13090791

**Published:** 2022-08-31

**Authors:** Zhong-Ping Hao, Lei Sheng, Zeng-Bei Feng, Wei-Xin Fei, Shu-Min Hou

**Affiliations:** Crop Research Institute, Anhui Academy of Agricultural Sciences, Hefei 230031, China

**Keywords:** cabbage aphid, electropenetrography, oilseed rape, probing behavior, transmission, turnip mosaic virus, vector

## Abstract

**Simple Summary:**

Winter oilseed rape accounts for about 90% of total oilseed rape planting area in China, with the majority of it concentrated in the Yangtze River basin. The remaining 10% is mostly found in the provinces of China’s northwest plateau. Winter oilseed rape areas in China have gradually expanded to the north in the last decade, resulting in cabbage aphids and turnip mosaic virus (TuMV). The aphid is a vector for TuMV and is gradually increasing on winter and spring oilseed rape. Quantifying the probing behaviors of the aphids on spring oilseed rape and winter oilseed rape helps us to understand TuMV regulation of the aphids. We found that compared to mock-inoculated plants, cabbage aphids on infected plants increased brief probing frequency, cell penetration frequency, intracellular probing time, decreased time to first probe and pathway duration, potentially promoting viral acquisition and minimizing viral loss and plant damage. Viruliferous aphids had reduced pathway duration, increased cell penetration frequency, increased intracellular probing time, increased salivation frequency, and ingested less sap compared with non-viruliferous aphids, primed for viral infection. TuMV infection also differentially modified aphid feeding behavior on winter and spring oilseed rape cultivars, primarily on uninfected plants.

**Abstract:**

Direct and indirect effects of plant virus infection on vector behavior have been discovered to improve virus transmission efficiency, but the impact of plant cultivars in virus–vector–plant interactions has received little attention. Electropenetrography (EPG) allows real-time tracking and quantification of stylet penetration behaviors, pathogen transmission, and plant resistance mechanisms. Quantitative probing behaviors on a spring oilseed rape cultivar, ‘Xinyou17’, and a winter oilseed rape cultivar, ‘Zheping4’, were investigated using EPG to compare turnip mosaic virus (TuMV) regulation of cabbage aphid probing behavior. Results for indirect effects showed that compared to mock-inoculated plants, cabbage aphids on infected plants increased brief probing frequency, cell penetration frequency, intracellular probing time, and decreased time to first probe and pathway time, potentially promoting viral acquisition. TuMV also directly influences aphid probing behavior. Viruliferous aphids had reduced pathway time, increased cell penetration frequency, increased intracellular probing time, increased salivation frequency, and ingested less sap than non-viruliferous aphids, primed for viral infection. Although oilseed rape cultivars can also influence aphid behavior, the main effect of cultivars was not significant on TuMV-infected plants.

## 1. Introduction

Plant virus infection changes both plant characteristics and vector (often insect) behavior, the changes of which in turn affect virus transmission mode and efficiency [1]. According to the ‘Vector Manipulation Hypothesis’, plant viruses can influence their vectors’ behavior and fitness in two ways: indirectly (via changes in the plant’s physiological and biochemical properties as a result of infection) and directly (via the presence of the virus in the vector’s body) [2,3,4].

Plant virus infection causes symptoms, which indirectly manipulate vector behavior in order to improve virus fitness [5,6,7]. Geminiviruses, for example, which are transmitted in a persistent and circulative manner, can cause leaf curl and folds, as well as changes in epidermal trichomes [8] and resistance inhibition of host plants [9,10], thereby attracting vector *Bemisia tabaci* Gennadius (Hemiptera: Aleyrodidae) and resulting in *B. tabaci* having better-balanced nutrient absorption, higher survival and oviposition rates, and a faster population growth rate than it does on healthy tobacco [11,12,13]. However, viruses transmitted in a non-persistent and non-circulative manner may alter host gustatory signals to repel vectors rather than encourage colonization [1]. *Aphis gossypii* Glover (Hemiptera: Aphididae) is initially attracted to cucumber mosaic virus (CMV) (Bromoviridae: Cucumovirus)-infected squash plants (*Cucurbita pepo* Linnaeus) but thereafter disperses to colonize virus-free plants preferentially. CMV infection increased the number of short superficial probes as well as the number of intracellular punctures, a behavioral pattern critical for non-persistent virus transmission [14]. Furthermore, in the second hour of recording, aphids spent considerably less time salivating and ingesting on CMV-infected plants than on mock-inoculated plants [4,15]. It appears that non-persistent viruses may indirectly alter insect vector behavior to maximize transmission by infecting plants [4].

Plant viruses can also directly modify vector feeding behavior to facilitate their transmission [2,5,12,15]. Begomoviruses, in a persistent and circulative mode of transmission, are acquired by *B. tabaci* as intact virions from plant phloem, which migrate down food canal, foregut and esophagus, and reach midgut, where they are absorbed into hemolymph via filter chamber [16]. Long-term viral interactions with *B. tabaci* can change vector physiological and biochemical properties, causing viruliferous whitefly adults to move at a slower rate than non-viruliferous whitefly adults [8,17]. Early research with potyviruses and cauliflower mosaic virus (CaMV) (Caulimoviridae: Caulimovirus), which are retained in the stylet or foregut and transmitted in a non-persistent and non-circulative manner, also demonstrated that aphid transmission of these viruses is caused by specific interactions rather than simply contamination of virions on aphid stylets. CaMV uses a helper strategy to achieve aphid transmission [18], which requires interactions between three CaMV-encoded proteins, one of which, CaMV-encoded P2, is a non-virion helper component protein that binds to the aphid stylet via its N-terminus while also binding to the N-terminal region of P3, which is anchored within the virion capsid shell via its C-terminus [19,20].

Turnip mosaic virus (TuMV) (Potyviridae: Potyvirus), as a member of the potyvirus family, is transmitted in aphid stylets through a non-persistent and non-circulative manner, and has been reported to infect oilseed rape *Brassica napus* L. in various growing areas around the world [21,22,23,24], resulting in a 70 to 79% yield reduction [22]. China’s oilseed rape production is divided into two major growing areas: winter and spring oilseed rape areas. Winter oilseed rape accounts for about 90% of total oilseed rape planting area in China, with the majority of it concentrated in the Yangtze River basin. Winter oilseed rape is typically sown in the autumn and harvested in the following summer. Spring oilseed rape planting area accounts for approximately 10% of total oilseed rape planting area. It is mostly found in the provinces of China’s northwest plateau. Spring oilseed rape is typically planted in the spring. Winter oilseed rape areas in China have gradually expanded to the north in the last decade, owing to successful breeding of strong cold-resistant winter oilseed rape cultivars and climate warming, resulting in many diseases and insect pests now occurring in both winter and spring oilseed rape. The geographic range and survival time of aphids, the main winter and spring oilseed rape pests, have increased, as has the corresponding spread of virus disease [25]. In major rapeseed producing areas in China, *Lipaphis erysimi* Kaltenbach and *Myzus Persicae* Sulzer were the primary TuMV vectors, but *Brevicoryne brassicae* L. (Hemiptera: Aphididae) has gradually increased and received more attention in recent years than the other two species [26,27]. In oilseed rape crops, an increase in viral symptoms (leaf mosaic, vein clearing, leaf and stem distortion, and growth reduction) associated with TuMV has also been observed. It is unclear, however, how TuMV infection affects aphid probing behavior to enhance their own transmission on spring and winter oilseed rape, and the illustration could lead to novel and broad-approaches to prevent TuMV transmission by cabbage aphids.

We evaluated the outcomes of three different treatments on spring and winter oilseed rape cultivars, demonstrating the host acceptance of cabbage aphids as well as the indirect and direct effects of TuMV infection on aphid probing behavior. To do this we used electropenetrography (EPG), which could accurately assess the acceptance of leaf surface, ease of stylet penetration, and degree of phloem acceptance among various oilseed rape cultivars [28,29]. TuMV infection could differentially modify aphid probing behavior on spring and winter oilseed rape cultivars, and non-viruliferous aphids might tend to acquire the virus on TuMV-infected plants, whereas viruliferous aphids may find it easier to inoculate the virus on uninfected plants.

## 2. Materials and Methods

### 2.1. Insects and Plants

*Brevicoryne brassicae*, cabbage aphids, were isolated from oilseed rape plants growing in the greenhouse at the Institute of Vegetables, Zhejiang Academy of Agricultural Sciences. The aphids were raised in a controlled growth chamber for one year on a *Brassica oleracea* var. *capitata* (L.) (Capparales: Brassicaceae) cultivar at 25 ± 1 °C, 75 ± 5% relative humidity (RH), and 16:8 (L:D) photoperiod. All of the aphids used in the study were descended from a single virginoparous apterous aphid. Individual aphids from this colony were used in experiments described below.

Two *Brassica napus* var. *napus* (L.) cultivars, spring oilseed rape cultivar ‘Xinyou17’ (Ogura cytoplasmic male sterility) and winter oilseed rape cultivar ‘Zheping4’ (Hybridization and systematic breeding from two autogamous inbred lines) were selected from the Laboratory of Plant Breeding of Anhui Academy of Agricultural Sciences, China. When the two cultivars were compared in the scientific research field of Anhui Academy of Agricultural Sciences in 2020, they both had a similar TuMV incidence (around 30%) (Unpublished results). Plants were grown in 13-cm diameter plastic pots with a mixture of peat moss, vermiculite, organic fertilizer (N + P_2_O_5_ + K_2_O 2%, organic matter 40%, Zhongnuo, Huaian, Jiangsu, China), and perlite (10:10:10:1 ratio). The plants were grown at 25 ± 1 °C, 75 ± 5% RH, and 12:12 (L:D) photoperiod, with regular watering and no additional fertilizer. TuMV inoculation or mock inoculation was performed on plants with two fully expanded leaves of each *B. napus* cultivar. These treated plants were used for electropenetrography (EPG) studies during the four-leaf stage, based on our previous research [28,30].

### 2.2. TuMV

TuMV-CRl, a mild strain, was the most widely disseminated line in China [31]. The Tobacco Research Institute, Anhui Academy of Agricultural Sciences, China, originally provided an isolate of this strain. Plants were inoculated at the 2-true leaf stage and used as viral sources after inoculation at the 4-true leaf stage.

### 2.3. TuMV Infection

Mechanical inoculation at the two-leaf stage with leaf sap from symptomatic leaves resulted in infected plants with four fully expanded leaves [32]. Mock-inoculated oilseed rape plants were rubbed with buffer solution (0.07 M potassium phosphate containing 0.02 M mercaptoethanol, pH 7.0) and quartz sand by a hand with a sterilized glove as non-infected controls. All of the plants were grown in an insect-proof chamber at 24:20 °C (D:N), a photoperiod of 16:8 h (L:D), and a relative humidity of 60–80%. Because there was no significant difference in aphid feeding behavior between mock-inoculated and uninoculated plants (data not shown), the EPG data from mock-inoculated plants were used as control data and were utilized to compare host acceptance by aphids between the two cultivars.

Half of the newly molted (2 days) apterous aphids were brushed onto bouquets of TuMV-infected oilseed rape leaves and the other half onto bouquets of mock-inoculated oilseed rape leaves to achieve two experimental treatments: viruliferous and non-viruliferous aphids. After a 2-h fast, the aphids were given a 5-min acquisition access period on the oilseed rape bouquets before being gently brushed off and used for EPG [33].

### 2.4. TuMV Identification

Infected oilseed rape plants were housed in an aphid-free glasshouse for 8 to 21 days after mechanical inoculation or aphid exposure, and virus symptoms were monitored daily. Each week, one hundred non-aphid-exposed plants in the glasshouse were also checked to ensure that no TuMV had been introduced. The presence of TuMV in symptomatic plants and aphids was confirmed using the double antibody sandwich enzyme linked immuno-sorbent assay DAS-ELISA method, which was performed with a turnip mosaic virus ELISA kit from SenBeiJia Biological Technology Co., Ltd., Nanjing, Jiangsu, China.

### 2.5. Response of Plants to TuMV Infection

The approach of Chen et al. (2006) [32] was used to identify plant response to TuMV infection in two oilseed rape cultivars. The study was carried out in an insect-proof chamber in the year 2020. Ten to fifteen plants of each cultivar were mechanically inoculated with TuMV at the 2-leaf stage. The inoculated plants were kept in the chamber for a month to track symptom progression. The disease index was calculated using TuMV infection incidence and severity. Each cultivar’s response was assessed using the average disease index (ADI) [32]. Based on ADI, the five levels of resistant (susceptible) response are immune: 0; high resistant response: 0.1–10.0; medium resistant response: 10.1–30.0; medium susceptible response: 30.1–50; and highly susceptible response: more than 50.

### 2.6. Microscopic Observation of Leaf Surface

Leaf properties were observed and investigated using the methodologies of Yan and Wang (2017) and Hao et al. (2019) [1,30]. Each cultivar’s uppermost first leaf was collected at the four-leaf stage. The obtained samples were prefixed with 2% glutaraldehyde overnight. The prefixed samples were washed in phosphate buffer (PBS, pH = 7.4) the next morning, then postfixed in 2% OsO4 for 1 h before being washed in PBS at pH 7.4. The samples were dehydrated in a series of gradient alcohol concentrations before embedding in Epon 812 resin (SPI Supplies, Structure Probe, Inc., West Chester, Pennsylvania, The United States of America). Following a semi-thin section (3–4 µm), the leaf structures were photographed using an inverted phase contrast microscope equipped with a Leica DFC 295 imaging system (Leica DM IRB, Leica Microsystems, Wetzlar, Germany). The thickness of the upper epidermis, as well as the quantity and length of trichomes, were measured using the Leica application suite (LAS, Leica Microsystems, Wetzlar, Germany). Ten leaves from ten plants were inspected per replicate for each cultivar, with three replicates carried out.

### 2.7. EPG Experiments

The EPG protocol described by Hao et al. (2019; 2020) [29,30] was used to track apterous adult aphids’ penetration activities on oilseed rape plants. The aphid electrode was connected to a four-channel DC-EPG system (Giga-4; EPG Systems, Wageningen, The Netherlands), and the EPG output was captured with PROBE 3.5 (hardware and software from EPG-Systems, Wageningen, The Netherlands). The EPG test was carried out on apterous adults who had just molted (2 days). Each cultivar was subjected to three treatments. EPG recordings were made on plants within a Faraday cage. Non-viruliferous aphids on mock-inoculated plants were included in Treatment I (negative control), non-viruliferous aphids on infected plants were included in Treatment II, and viruliferous aphids on mock-inoculated plants were included in Treatment III. The tethered aphid was placed on the upper side of the mature leaf midrib of the test plant quickly (<30 min after being collected from the rearing plant). In a laboratory setting with constant lighting and a temperature of 25 ± 1 °C, each treatment was recorded over thirty times. Aphids and plants were used only once per recording. The feeding behavior of *B. brassicae* was monitored for 6 h. EPG profiles were recorded with an A/D card (DI-710 format, Dataq Instruments Incorporated, Akron, OH, USA) and analyzed with the Stylet+ software. The waveform definitions recorded in EPG analyses for each aphid were listed in Table 1, and the data were automatically analyzed using the MS Excel workbook for automatic parameter calculation of EPG data (version 4.4, Madrid, Spain) developed by Sarria et al. (2009) [34]. Any aphid that did not probe within 10 min of being recorded was discarded.

### 2.8. Statistics Analyses

Data were transformed (square-root transformation for the number of occurrences, natural log transformation for the duration, and square-root arcsine transformation for the proportion) and analyzed using SAS 9.2 software (SAS Institute Inc., Cary, NC, USA). In this study, the relative acceptance to aphids and the differences in response to TuMV infection were compared between the two oilseed rape cultivars, and the differences of EPG variables between treatment I (negative control) (feeding behavior of non-viruliferous aphids on uninfected plants) and treatment II (feeding behavior of non-viruliferous aphids on infected plants) and between treatment I and treatment III (feeding behavior of viruliferous aphids on uninfected plants) were compared on the two cultivars, respectively, using Mann–Whitney U-test (for non-Gaussian variables) or Student’s *t*-test (for Gaussian variables) [28,30,35]. The significance of the main effects of cultivar and infection status as well as their interaction were analyzed using two-way ANOVA. *p* = 0.05 was chosen as the significance level. Despite the fact that all statistics were calculated on all variables, only the essential variables are shown in the tables and figures.

## 3. Results

### 3.1. Response of Plants to TuMV Infection

The average disease incidence and average disease index were not different between the two cultivars, and the data for the two cultivars fell within the medium TuMV resistant response range (Table 2).

### 3.2. Characteristics of Leaf Surface

Leaf properties of the two cultivars differed (Table 3). ‘Xinyou17’ had a thicker upper epidermis (*F* = 2.32, *p* < 0.01) and fewer trichomes than ‘Zheping4’. The trichome length of the two cultivars did not differ statistically (Table 3).

### 3.3. Treatment I: EPG Revealing Host Acceptance of Cabbage Aphids on Two Oilseed Rape Cultivars

**Figure 1:**
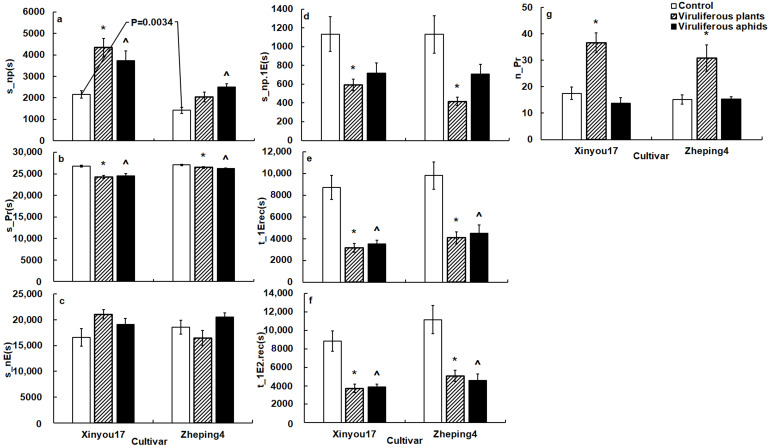
The general variables of *B. brassicae* probing behavior on the two oilseed rape cultivars in the TuMV-infected and mock-inoculated treatments. The *p* value on the white columns indicates a statistical difference between the two cultivars; (**a**), the impact of TuMV infection on total non-probing time of aphids; (**b**), the impact of TuMV infection on total probing time of aphids; (**c**), the impact of TuMV infection on total no phloematic duration of aphids; (**d**), the impact of TuMV infection on non-probing duration before the first phloem contact of aphids; (**e**), the impact of TuMV infection on time from the start of EPG to the first phloem contact of aphids; (**f**), the impact of TuMV infection on time from the start of EPG to the first ingestion of aphids; (**g**), the impact of TuMV infection on probing frequency of aphids; * on the gray columns denotes a statistical difference between infected plant and mock-inoculated control in the same cultivar; ^ on the black columns indicates a statistical difference between viruliferous aphids and non-viruliferous aphids in the same cultivar. The acronym of the variables is defined in Table 1.

There were a few variables that reflected host acceptance with statistical differences in different tissue levels (Figure 1, Table 4).

- General EPG variables. Aphids spent significantly less non-probing time (s_np) (*F* = 1.15, *p* = 0.0034) on ‘Zheping4’ than on ‘Xinyou17’ (Figure 1a). Other general variables between the two cultivars revealed no significant differences (Figure 1b–g).
- Surface-mesophyll EPG variables. Aphids started their first probe much later on ‘Xinyou17’ than they did on ‘Zheping4’. Aphids penetrated cells later and produced fewer intracellular punctures in the mesophyll of ‘Xinyou17’ than in that of ‘Zheping4’. Aphids on ‘Xinyou17’ had a shorter pathway duration and spent a smaller percentage of their probing time in the pathway than on ‘Zheping4’ (Table 4).
- Phloem EPG variables. Aphids on ‘Xinyou17’ had significantly higher values associated with ingestion, such as s_longestE2, %sE2/E2, and %probtiminE2 than on ‘Zheping4’. Aphids in the phloem of ‘Xinyou17’, on the other hand, had a lower salivation frequency, spent less time salivating, had a shorter salivation time before the first sustained ingestion, secreted less saliva on periods of salivation and ingestion, and exhibited a lower percentage of probing in salivation than in the phloem of ‘Zheping4’ (Table 4).

Based on EPG and microscopic data, ‘Zheping4’ relative impediments appeared to be located in mesophyll and phloem, whereas ‘Xinyou17’ relative impediments were located at the leaf surface level (Table 4).

### 3.4. Treatment II: TuMV Indirectly Modifying Cabbage Aphid Probing Behavior by Infecting Oilseed Rape

- General EPG variables. TuMV infection substantially altered aphid probing behavior. Cabbage aphid probing behavior differed between TuMV-infected ‘Xinyou17’ and TuMV-infected ‘Zheping4’ (Figure 1).

Aphids significantly reduced total probing time, non-probing time before the first contact with phloem, duration before the first phloem contact and before the first ingestion, and increased probing frequency on infected plants when compared to mock-inoculated plants (Figure 1b,d–g). Aphids did not significantly change the total duration of the no phloematic phase on infected plants compared to mock-inoculated plants (Figure 1c).

Aphids spent much more non-probing time on infected ‘Xinyou17’ than on mock-inoculated plants, though aphids on infected ‘Zheping4’ did not show significant difference in s_np between infected and mock-inoculated plants (Figure 1a).

- Surface EPG variables. Aphids began penetrating the leaf surface on infected plants earlier than on mock-inoculated plants (Table 4).
- Mesophyll EPG variables. Aphids significantly reduced the time from the start of that probe to the first phloem contact on TuMV-infected plants. In comparison to mock-inoculated plants, aphids on infected plants started initial cell penetration earlier, tasted in mesophyll cells for a longer period, and significantly increased the frequency of brief probes and cell penetration (Table 4).

Aphids reduced pathway duration on infected ‘Zheping4’, compared to mock-inoculated plants, but not on infected ‘Xinyou17’ (Table 4).

When compared to mock-inoculated plants, aphids significantly increased the proportion of time for pathway duration on infected ‘Xinyou17’, but reduced the proportion on infected ‘Zheping4’ (Table 4).

- Phloem EPG variables. Aphids on infected plants significantly increased salivation frequency. On TuMV-infected plants, aphids reduced the duration of salivation followed by the first sustained ingestion, and required less salivation duration before sustained ingestion. Aphids contributed less sustained ingestion time to the ingestion phase on infected plants than mock-inoculated plants (Table 4).

Aphids spent significantly less time in ingestion and longest ingestion on ‘Xinyou17’ after TuMV infection than mock-inoculated plants, and had a lower ingestion index and a lower percentage of ingestion, but contributed a significantly higher percentage of salivation phase to complete probing, spent a longer salivation phrase, and contributed a higher rate of salivation to phloem phase on infected ‘Xinyou17’ than mock-inoculated plants (Table 4). Aphids on ‘Zheping4’ revealed no significant differences in these variables between infected and mock-inoculated plants.

### 3.5. Treatment III: TuMV Directly Modifying Cabbage Aphid Probing Behavior by Infecting Aphids

- General EPG variables. In comparison to non-viruliferous aphids, viruliferous aphids spent much less time in the total probing phase and in approaching phloem and sieve elements from EPG start (Figure 1b,e,f). However, viruliferous aphids spent significantly more non-probing time than non-viruliferous aphids (Figure 1a). Other general variables between viruliferous and non-viruliferous aphids revealed no significant differences (Figure 1c,d,g).
- Surface EPG variables. Viruliferous aphids penetrated leaves later than non-viruliferous aphids on ‘Zheping4’ but showed no significant difference on ‘Xinyou17’ from non-viruliferous aphids (Table 4).
- Mesophyll EPG variables. Viruliferous aphids took less time to reach phloem (t_1EinPr) than non-viruliferous aphids. Viruliferous aphids tasted in cells significantly earlier than non-viruliferous aphids. Viruliferous aphids tasted longer in mesophyll cells than non-viruliferous aphids. Viruliferous aphids produced more punctures in cell than non-viruliferous aphids (Table 4). The difference of %probtimeinC between viruliferous and non-viruliferous aphids was not significant (Table 4).

When compared to non-viruliferous aphids, viruliferous aphids reduced brief probing frequency on ‘Xinyou17’. However, viruliferous aphids showed no significant differences in the variable on ‘Zheping4’ when compared to non-viruliferous aphids (Table 4).

When compared to non-viruliferous aphids on ‘Zheping4’, viruliferous aphids reduced the pathway time. Viruliferous aphids did not differ significantly from non-viruliferous aphids on ‘Xinyou17’ (Table 4).

- Phloem EPG variables. Viruliferous aphids secreted saliva more frequently than non-viruliferous aphids. Viruliferous aphids, on the other hand, spent less time secreting saliva before the initial sustained ingestion than non-viruliferous aphids. In the phloem, viruliferous aphids spent significantly less time in longest ingestion, and had a lower proportion of sustained ingestion to ingestion phrase and a lower ingestion index than non-viruliferous aphids (Table 4).

In comparison to non-viruliferous aphids, viruliferous aphids required a lower percentage of ingestion but contributed a higher rate of salivation to the phloem phase, provided a higher proportion of salivation to total probing, spent a longer salivation phrase, and required more salivation duration before sustained ingestion in the phloem of ‘Xinyou17’, while the variables were equivalent in the phloem of ‘Zheping4’ (Table 4).

### 3.6. Analysis of Cultivar and TuMV Infection Main Effects

#### 3.6.1. TuMV Infecting in Plants

**Figure 2:**
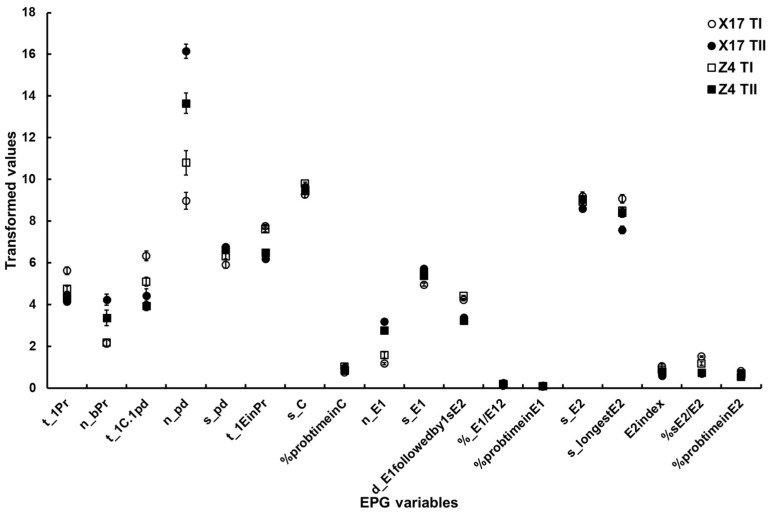
The major EPG variables of *B. brassicae* probing behavior on the two oilseed rape cultivars with different TuMV infection status. X17 T1 represents treatment I (feeding behavior of non-viruliferous aphids on uninfected plants) on Xinyou17; X17 TII represents treatment II (feeding behavior of non-viruliferous aphids on infected plants) on Xinyou17; Z4 T1 represents treatment I (feeding behavior of non-viruliferous aphids on uninfected plants) on Zheping4; Z4 TII represents treatment II (feeding behavior of non-viruliferous aphids on infected plants) on Zheping4. A summary of the two-way ANOVA, comparing the major EPG variables of aphid probing behavior between the two cultivars with different TuMV infection status, is presented Table 5. The acronym of the variables is defined in Table 1.

- Surface EPG variables. Cultivars and TuMV infection status had a significant impact on the t_1Pr variable, and there was also a significant interaction between the two factors.
- Mesophyll EPG variables. The two-way ANOVA suggested significant differences in n_bPr and t_1EinPr depending on the TuMV infection status, but it did not reveal a significant difference between the two cultivars or a significant interaction between the two factors. Both cultivars and TuMV infection status significantly affected the t_1C.1pd variable, but there was no evidence of a significant interaction between the two factors. There were significant differences in n_pd and s_pd between TuMV infection status, but not between the two cultivars. Significant interactions existed between the two factors. Although cultivars and TuMV infection status did not significantly differ in s_C and %probtimeinC, significant interactions between the two factors were discovered.
- Phloem EPG variables. Except for s_E2 and %probtimeinE2, significant differences between TuMV infection status could be found for the majority of phloem EPG variables. Only %sE2/E2 was significantly different between the two cultivars. All of the phloem EPG variables showed significant interactions between the two factors (Table 5, Figure 2).

#### 3.6.2. TuMV Vectored in Aphids

**Figure 3:**
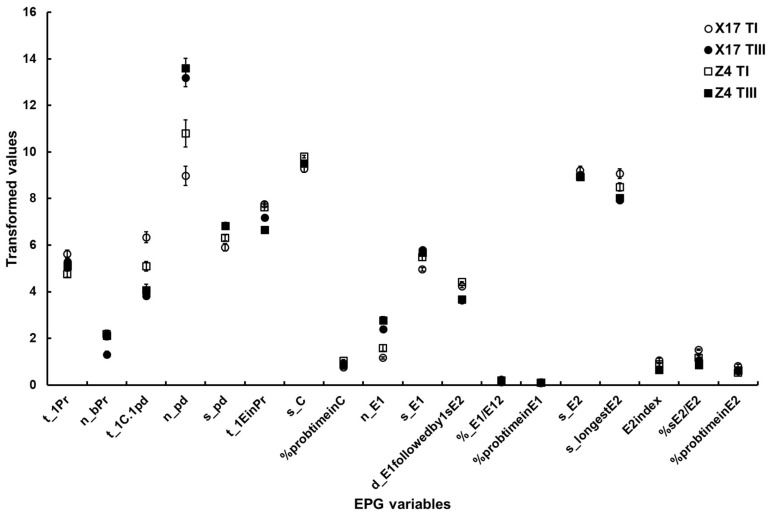
The major EPG variables of *B. brassicae* probing behavior with different TuMV infection status on the two oilseed rape cultivars. X17 T1 represents treatment I (feeding behavior of non-viruliferous aphids on uninfected plants) on Xinyou17; X17 TIII represents treatment III (feeding behavior of viruliferous aphids on uninfected plants) on Xinyou17; Z4 T1 represents treatment I (feeding behavior of non-viruliferous aphids on uninfected plants) on Zheping4; Z4 TIII represents treatment III (feeding behavior of viruliferous aphids on uninfected plants) on Zheping4. A summary of the two-way ANOVA, comparing the major EPG variables of aphid probing behavior with different TuMV infection status between the two cultivars, is presented in Table 5. The acronym of the variables is defined in Table 1.

- Surface EPG variables. The two-way ANOVA showed a significant difference in t_1Pr between the two cultivars, but did not show a significant difference between TuMV infection status. However, a significant interaction between the two factors was discovered.
- Mesophyll EPG variables. Although there were significant differences in n_bPr and n_pd both between cultivars and between TuMV infection status, no significant interactions between the two factors were found. There were significant differences in t_1EinPr between cultivars, and between TuMV infection status, and a significant interaction between the two factors. A significant difference in s_pd between TuMV infection status was showed, but no significant differences between the two cultivars and interactions between the two factors were found. There was a significant difference in t_1C.1pd between TuMV infection status, but not between cultivars. Additionally, a significant interaction between the two factors was discernible. There were significant differences in s_C and %probtimeinC between cultivars, but there were no significant differences between TuMV infection status. However, there were significant interactions between the two factors.
- Phloem EPG variables. Between cultivars and between TuMV infection status, significant differences in n_E1, %sE2/E2 and d_E1 followedby1sE2 could be found, but no significant interactions between the two factors could be found. The two-way ANOVA suggested significant differences in %probtimeinE1 and E2index between TuMV infection status but not between the two cultivars, and it did not suggest significant interactions between the two factors. Significant differences in s_E1, %_E1/E12 and s_longestE2 between TuMV infection status were detected, but not between cultivars. Significant interactions between the two factors could be found. There were no significant differences in %probtimeinE2 between cultivars or TuMV infection status, but there was a significant interaction between the two factors (Table 5, Figure 3).

## 4. Discussion

### 4.1. Host Acceptance

Spring oilseed rape cultivar ‘Xinyou17’ had fewer trichomes than winter oilseed rape cultivar ‘Zheping4’. Surface characteristics on stems and leaves, such as trichomes or spines, differ between cultivars and can influence aphid settling, feeding, mating, and oviposition [36]. Aphids on the leaf upper surface of ‘Xinyou17’ delayed the first probe significantly more than ‘Zheping4’, implying that the hindrance factors may be located in the surface cell layer of the leaves of ‘Xinyou17’ or the attractive factors in that of ‘Zheping4’. Plant volatiles can influence the time an aphid takes from being placed on the plant until first probe [30]. However, most of the current studies are mainly based on indirectly inferred conclusions from the results of the attraction or repulsion of plant volatiles to insects in olfactory studies combined with EPG results, and few studies have directly investigated the effects of volatiles on EPG variables. In the few known studies involving both volatiles and EPG variables, plant volatiles were found to affect the time to first probe, such as the study of Ninkovic et al. (2021) [37], which reported that exposure to methyl salicylate results in a longer time from the start of the EPG recording until the first probe of *Rhopalosiphum* *padi* (L.) on exposed barley plants compared to unexposed plants at day 3. The volatiles of the two cultivars in our study may differ, leading to different attractive to aphids; therefore, further studies will be required.

Aphids in the mesophyll of spring oilseed rape cultivar ‘Xinyou17’delayed penetrating cells, produced fewer intracellular punctures, and had a shorter pathway duration than aphids in the mesophyll of winter oilseed rape cultivar ‘Zheping4’. In order to navigate to sieve elements, an aphid’s stylet punctures almost every cell along the stylet pathway [38]. Any intercellular or intracellular components in mesophyll that prevent the aphid stylet from entering the phloem could lengthen the pathway phase. ‘Zheping4’ mesophyll might contain intracellular obstructive factors.

Aphid feeding in phloem will be necessarily changed as the food supply shifts. Plant resistance or susceptibility indices are typically provided as E1 (representing salivation) and E2 (indicating ingestion) [39]. Cabbage aphids secreted more saliva and ingested less sap on ‘Zheping4’ than on ‘Xinyou17’. It had been proposed that aphids on ‘Zheping4’ would be confronted with plant defenses, feeding deterrents and/or inadequate nutritional content in the phloem sap [40,41]. We discovered that some factors in the leaf surface level of ‘Xinyou17’ and some in the mesophyll and phloem levels of ‘Zheping4’ inhibited aphid feeding behavior based on EPG and electron microscopy results.

### 4.2. Indirect Effects of TuMV Infection

Plant virus infection, such as TuMV in our study, could alter host plant suitability for aphid vectors and influence aphid vector behavior [6,42,43,44]. Following TuMV infection in the two oilseed rape cultivars, cabbage aphids significantly reduced probing time per probe and the time outside the first phloem contact. On infected spring oilseed rape cultivar, meanwhile, ‘Xinyou17’ significantly increased non-probing time, implying that aphids might spend less pathway duration and phloem phase on infected ‘Xinyou17’ than on mock-inoculated plants. It denoted a weakened impediment in the infected mesophyll and an increased impediment in the infected phloem of ‘Xinyou17’.

Cabbage aphids on infected plants penetrated the leaves earlier than on mock-inoculated plants, indicating that TuMV infection in oilseed rape reduced the impediment or enhanced the attraction to aphids at the leaf surface level. In the systems of wheat-barley yellow dwarf virus (BYDV) (Luteoviridae: genus Luteovirus)-*Rhopalosiphum padi* L. and potato-potato leafroll virus (PLRV) (Luteoviridae: Polerovirus)-*M. persicae* S. [42,44], aphids preferentially colonized virus-infected plants because infected tissues were yellow, making them more visually appealing to aphids. However, when aphids are prevented from reaching the leaf surface or when visual cues are absent, this preference remains [45,46], indicating that olfactory cues may be more important than visual cues in mediating such responses [46,47]. Plant volatiles released by CMV-infected plants attracted aphids exclusively during the early stages of aphid-plant contact, according to Carmo-Sousa et al. (2014) [4] and Mauck et al. (2010) [15]. TuMV infection may have altered the volatile organic compounds (VOC) of oilseed rape to attract aphids, most likely by reducing total volatile emission or terpenoids release [48,49]. According to the model of non-persistent virus transmission, the number of plants visited each day is a critical variable driving virus epidemics; therefore, this attraction of infected plants to aphid vectors could have significant implications for virus spread [50,51].

In comparison to mock-inoculated oilseed rape, cabbage aphids on infected plants significantly reduced the time between the start of that probe and the first phloem contact, which may have been responsible for effectively vectoring viral particles. The findings supported the above hypothesis, indicating a weakened barrier in the infected mesophyll. TuMV infection, on the other hand, resulted in more brief probes and intracellular punctures, as well as more time in penetrating cells than on mock-inoculated plants, which was comparable to CMV, another non-persistent virus. CMV also causes an increase in the number of short probes and intracellular punctures, and the number of penetrating cells in infected plants was found to be positively related to the efficiency of aphids acquiring viruses [4].

Long feeding probes are known to reduce transmission efficiency [52]. Previous studies on the non-persistent potyvirus Zucchini yellow mosaic virus (ZYMV, Potyviridae: Potyvirus) found that winged *A. gossypii* spent less time feeding in infected phloem than uninfected plants [6]. According to our findings, cabbage aphids on infected plants had a lower contribution of sustained ingestion to the ingestion phase and secreted less saliva before sustained ingestion than mock-inoculated plants. Non-persistent viruses may be able to stimulate the synthesis of chemical substances that prevent aphids from ingesting phloem sap over time [4]. Aphids declined significantly relevant variables associated with ingestion in infected ‘Xinyou17’ but not in infected ‘Zheping4’, implying that the phloem blockage induced by TuMV infection was stronger in ‘Xinyou17’ than in ‘Zheping4’. This conclusion was supported by higher salivation on ‘Xinyou17’ than on ‘Zheping4’. It suggested that the virus could make ‘Xinyou17’ phloem more unsuitable to aphids, reducing aphid feeding and minimizing viral loss.

According to Carmo-Sousa et al. (2014) [4], viruses transmitted in a non-persistent manner may follow the general rule that an increase in the frequency of brief superficial probes and intracellular punctures followed by a phloem-feeding discouragement improves virus transmission efficiency. On infected plants, we observed increased intracellular penetration frequency and time, increased salivation, and hampered ingestion. The rule was thus validated on the two oilseed rape cultivars in our study, and it was more applicative in spring oilseed rape cultivars than in winter oilseed rape cultivars.

### 4.3. Direct Effects of TuMV Infection

Non-viruliferous vectors tend to feed on infected plants, whereas viruliferous vectors, such as BYDV-vectoring *R. padi* L. [3,53], tend to feed on uninfected plants [54]. The preference emerges only at the start of the viruliferous aphid-plant interaction. Viruliferous aphids spent much more time non-probing, less time probing, and less time beyond the first phloem contact than non-viruliferous aphids, indicating that viruliferous aphids reduced the pathway duration from the epidermis to the first phloem contact. The fact that viruliferous aphids had a longer total non-probing duration than non-viruliferous aphids suggested that the attraction of mock-inoculated plants to viruliferous aphids was weakened at later stages of recording.

According to Ingwell et al. (2012) [3] and Medina-Ortega et al. (2009) [47], the responsiveness of viruliferous aphids to host plant volatiles or other plant characteristics differs from that of non-viruliferous aphids. Viruliferous *M. persicae*, for example, is less susceptible to host VOC than non-viruliferous *M. persicae* [55]. Similarly, viruliferous aphids delayed first probing on ‘Zheping4’ but not on ‘Xinyou17’ when compared to non-viruliferous aphids. The attraction of VOC on the leaf surface of ‘Zheping4’ to viruliferous aphids might be reduced in this study. As a result, more research into the VOCs differences between the two cultivars is needed.

Reduced pathway duration and increased short probes and intracellular punctures have a positive link with the acquisition and subsequent inoculation of non-persistent viruses because aphids must release viruses to live cells to ensure the survival of viral particles [56,57] such as CMV, the inoculation efficiency of which was found to be positively linked with sub-phase pd II-2 produced viruliferous cotton aphids [58]. Similarly, viruliferous cabbage aphids reduced pathway duration prior to initial phloem contact, started penetrating cells earlier, produced more cell punctures, and spent much more time penetrating cells than non-viruliferous aphids, all of which may be conducive to viral inoculation. The shorter pathway duration of viruliferous aphids suggested that they prefer mock-inoculated plants over non-viruliferous aphids.

Short- and long-ingestion probes are known to be damaging to plant tissues and thus unlikely to aid virus transmission [4,59]. He et al. (2015) [60] discovered that the average feeding time of *B. tabaci* vectoring TYLCCNV on cotton was only approximately one-third that of non-viruliferous whiteflies, and that the virus had a negative impact on whitefly feeding behavior, but the negative impact could aid viral transmission. Wan et al. (2015) [61] discovered, using EPG, that RSV infection decreased the total time of phloem nutrient feeding while increasing the total time of saliva secretion during feeding in eclosion *L. striatellus* males compared to those who were not infected. Our results also showed that viruliferous aphids increased salivation frequency and decreased ingestion when compared to non-viruliferous aphids. As a result, TuMV infection may change directly vector preference for probing but not favor feeding. Meanwhile, viruliferous aphids significantly secreted more saliva than non-viruliferous aphids on ‘Xinyou17’ but not on ‘Zheping4’, implying that the direct modification for aphid feeding behavior in ‘Xinyou17’ may be more obvious than in ‘Zheping4’. TuMV compelled aphids to secrete more saliva and ingest less sap in order to improve transmission efficiency and reduce damage caused by aphid ingestion because the aphid-susceptible phloem of ‘Xinyou17’ is suitable for aphid feeding.

### 4.4. Analysis of Cultivar and TuMV Infection Main Effects

Based on the results of the two-way ANOVA (Table 5, Figure 2 and Figure 3), TuMV infection had a significant effect on aphid feeding behavior in all tissue levels of the leaves. For most EPG variables, the main effect of cultivars was non-significant. However, the interactions between cultivars and TuMV infection status were significant, implying that the effect of cultivars on aphid feeding behavior was also present and dependent on the effect of TuMV infection status. Among the variables associated with non-persistent virus acquisition—such as frequency and time of intracellular puncture, duration of the pathway period, and those related to salivary secretion and feeding—the interactions between cultivars and TuMV infection status were significant. This also indicated that TuMV infection in plants significantly altered aphid feeding behavior regardless of the cultivars, but that the effect of the virus infection differed significantly on different cultivars.

After aphids vectored the virus, there were significant interactions between virus infection status and cultivars on leaf surface-epidermis-related variables, pathway-related variables, and salivary secretion and the longest feeding duration in the phloem, indicating that the feeding behavior of viruliferous aphids was affected by the virus infection and cultivars compared to non-viruliferous aphids. However, the variables associated with virus inoculation, such as duration of intracellular puncture, were only affected by virus infection. Other variables associated with virus inoculation, such as frequency of short probes and frequency of intracellular puncture, were affected by both cultivars and virus infection without significant interactions. Rajabaskar et al. (2013) [62] reported that the cultivar effect and the interaction between potato cultivars and infection status were not significant in settling behavior of *M. persicae* on potato leafroll virus (PLRV)-infected and sham-inoculated plants. The effect of PLRV infection on settling behavior was significant for two of the cultivars, Desiree and Russet Burbank, whereas the difference was slight and non-significant for another two of the cultivars, Chipeta and IdaRose. This implied that the effect of cultivars as well as virus infection status on vector behavior is different in different vector–virus–plant systems. In our study, only two oilseed rape cultivars were selected, a spring rape cultivar ‘Xinyou17’ and a winter rape cultivar ‘Zheping4’, on which non-significant effects of cultivars on some variables were found (Table 5), suggesting that more cultivars should be selected to determine the differential effects of virus infection on vector probing behavior on different cultivars.

## 5. Conclusions

Virus-infected plants attract vector insects, and after acquiring viruses, vectors prefer healthy plants, resulting in virus transmission. As a result, viruses clearly influence vector probing behavior [63]. Our findings support the hypothesis that changing vector feeding behavior is a property shared by vector-transmitting plant viruses that has evolved as a mechanism to improve virus transmission [60,64]. TuMV’s effect on cabbage aphid feeding behavior, on the other hand, can be influenced by oilseed rape cultivars, and the influence was primarily reflected when plants were not infected by TuMV. As a result, TuMV incidence may not have differed significantly between the two cultivars. More representative cultivars with different genetic diversity should be selected, and the differences in volatile organic compounds among cultivars, and between infected and uninfected plants by TuMV, and the response of viruliferous and non-viruliferous aphids to the volatile organic compounds should be examined to further clarify these intricate linkages.

## Figures and Tables

**Table 1:** The definitions of the waveforms scored in the electropenetrography (EPG) analyses.

Acronym ^1^	Variable Type (Unit)	Definition
General		
n_Pr	Frequency	Number of probes
s_Pr	Time (s)	Total probing time
s_nE	Time (s)	Total duration of the no phloematic phase
s_np	Time (s)	Total time of the non-probing intervals
s_np.1E	Time (s)	Duration of the nonprobe period before the 1st E
t_1E2rec	Time (s)	Time from the start of EPG to the 1st E2
t_1Erec	Time (s)	Time from the start of EPG to the 1st E
Surface-mesophyll (Leaf)		
t_1Pr	Time (s)	Time to the first probe from the start of EPG
n_bPr	Frequency	Number of short probes (C < 3 min)
t_1C.1pd	Time (s)	Time from the beginning of the 1st probe to the first pd
n_pd	Frequency	Number of pd
s_pd	Time (s)	Total duration of pd
t_1EinPr	Time (s)	Time from the beginning of that probe to the 1st E
s_C	Time (s)	Total C duration with pd
%probtimeinC	Index (%)	% of probing spent in C
Phloem		
n_E1	Frequency	Number of E1 periods
s_E1	Time (s)	Total duration of E1
d_E1followedby1sE2	Time (s)	Duration of the E1 followed by the first sustained E2 (>10 min)
s_E1followedbysE2	Time (s)	Total duration of E1 followed by sustained E2 (>10 min)
%_E1/E12	Index (%)	Relative amount of E1 on E12
s_E2	Time (s)	Total duration of E2 periods
s_longestE2	Time (s)	Duration of the longest E2
E2index	Index (%)	phloemian index: % of the time of the E2 after the start of the 1st E2
%sE2/E2	Index (%)	Relative amount of sE2 on E2
%probtimeinE1	Index (%)	% of probing spent in E1
%probtimeinE2	Index (%)	% of probing spent in E2

^1^ All of the variables were analyzed within 6 h.

**Table 2:** The plant response to TuMV infection of two *B. napus* cultivars.

Cultivar ^1^	Average Disease Incidence%	Average Disease Index%	Response Rank
Xinyou17	25.80 ± 2.46	25.78 ± 2.59	medium resistant response
Zhongshuang11	28.30 ± 2.35	26.95 ± 2.94	medium resistant response

^1^ The values in the table show means±standard error (SE). The data were compared student’s *t*-test after square-root arcsine transformation. The level for significance was set to *p* < 0.05.

**Table 3:** Leaf properties of two oilseed rape cultivars.

Variable ^1^	Cultivar	
	Xinyou17	Zheping4
Thickness of upper epidermis (μm)	20.73 ± 0.59	14.43 ± 0.45 *
Trichome length (μm)	717.19 ± 15.68	712.41 ± 25.66
Trichome density on upper surface	11.73 ± 0.55	19.77 ± 1.64 *
Trichome density on lower surface	20.40 ± 0.56	28.37 ± 2.87 *

^1^ The values in the table show means±standard error (SE). The data were compared student’s *t*-test or Mann-Whitney U-test. The level for significance was set to *p* < 0.05.* within a row represents a statistical difference between the two cultivars.

**Table 4:** The main variables with significant difference between two oilseed rape cultivars.

Main Variables Associated with Behavior Modification ^1^	Cultivar					
	Xinyou17			Zheping4		
	Treatment I	Treatment II	Treatment III	Treatment I	Treatment II	Treatment III
Epidermis						
t_1Pr(s)	354.59 ± 53.85	71.21 ± 6.84	——	141.20 ± 23.67	78.38 ± 2.41	181.55 ± 22.90
Mesophyll						
n_bPr	——	19.25 ± 2.36	2.10 ± 0.36	——	13.90 ± 2.52	——
n_pd	83.65 ± 6.02	262.95 ± 10.79	176.75 ± 10.35	122.95 ± 11.61	190.95 ± 13.58	188.50 ± 11.86
s_pd(s)	——	905.81 ± 55.14	965.71 ± 52.27	——	796.39 ± 57.75	946.25 ± 45.26
t_1C.1pd(s)	782.10 ± 107.88	180.94 ± 42.76	52.99 ± 5.80	224.54 ± 37.77	71.94 ± 10.44	96.82 ± 20.63
t_1EinPr(s)	——	577.99 ± 74.32	1531.73 ± 184.41	——	752.92 ± 98.10	806.23 ± 51.17
s_C(s)	12,462.70 ± 1423.51	15,755.73 ± 881.24	——	18,638.07 ± 976.35	13,931.96 ± 1332.84	13,895.55 ± 745.90
%probtimeinC	46.11 ± 6.11	69.64 ± 3.41	——	70.23 ± 4.82	55.22 ± 5.17	——
Phloem						
n_E1	1.40 ± 0.11	10.3 ± 0.60	6.05 ± 0.58	2.90 ± 0.44	8.10 ± 0.83	8.20 ± 0.83
s_E1(s)	149.24 ± 10.89	333.12 ± 28.88	359.58 ± 33.15	301.16 ± 46.53	——	——
%_E1/E12	1.62 ± 0.35	6.39 ± 0.80	5.67 ± 0.81	4.29 ± 0.73	——	——
d_E1followedby1sE2(s)	70.17 ± 3.32	30.23 ± 1.87	38.27 ± 1.58	83.18 ± 2.00	26.49 ± 1.63	39.71 ± 1.22
s_E1followedbysE2(s)	——	83.33 ± 4.42	172.37 ± 16.69	——	65.88 ± 6.27	——
%probtimeinE1	0.57±0.04	1.43 ± 0.10	1.57 ± 0.16	1.04 ± 0.18	——	——
s_E2(s)	——	6714.86 ± 944.54	——	——	——	——
s_longestE2(s)	11,712.43 ± 1711.32	2670.77 ± 485.07	2937.37 ± 192.76	6129.23 ± 881.79	——	3718.66 ± 516.96
E2index	——	32.47 ± 4.14	39.88 ± 4.30	——	——	37.08 ± 3.62
%sE2/E2	98.11 ± 0.98	41.90 ± 5.24	68.23 ± 5.54	75.33 ± 5.46	46.57 ± 4.26	56.31 ± 3.09
%probtimeinE2	52.84 ± 6.13	28.64 ± 3.42	33.29 ± 3.86	29.90 ± 5.44	——	——
Response assessment	Relative mesophyll and phloem preference of aphids	TuMV-medium resistant response		Relative epidermis preference of aphids	TuMV-medium resistant response	

^1^ The values in the table show means±standard error (SE). The data were compared student’s *t*-test or Mann-Whitney U-test after square-root transformation for the number of occurrences, natural log transformation for the duration, and square-root arcsine transformation for the proportion. The level for significance was set to *p* < 0.05. —— indicates no statistical difference in treatment I between the two cultivars, or between treatment I and treatment II/treatment III in one cultivar.

**Table 5:** The main effect analysis of major EPG variables in different levels of leaves.

Variable	Treatment ^1^	Cultivar Main Effects		TuMV Infection Main Effects		Cultivar and TuMV Infection Interaction	
		*F*	*P*	*F*	*P*	*F*	*P*
Surface-mesophyll (Leaf)							
t_1Pr	Plant	5.13	**0.0267**	42.67	**0.0001**	16.28	**0.0001**
	Aphid	11.92	**0.0009**	0.002	0.9690	4.85	**0.0307**
n_bPr	Plant	1.84	0.1792	32.37	**0.0001**	2.15	0.1474
	Aphid	4.17	**0.0450**	6.15	**0.0156**	3.55	0.0639
t_1C.1pd	Plant	10.19	**0.0021**	27.58	**0.0001**	1.28	0.2623
	Aphid	3.20	0.0781	57.31	**0.0001**	11.31	**0.0013**
n_pd	Plant	0.28	0.5976	101.31	**0.0001**	17.60	**0.0001**
	Aphid	5.86	**0.0182**	51.20	**0.0001**	1.71	0.1956
s_pd	Plant	1.11	0.2966	22.68	**0.0001**	5.31	**0.0243**
	Aphid	2.85	0.0957	38.90	**0.0001**	3.56	0.0636
t_1EinPr	Plant	0.70	0.4052	107.06	**0.0001**	1.49	0.2264
	Aphid	4.61	**0.0357**	39.26	**0.0001**	4.13	**0.0462**
s_C	Plant	2.71	0.1045	−0.001	0.9741	13.28	**0.0005**
	Aphid	8.29	**0.0053**	0.31	0.5809	9.64	**0.0028**
%probtimeinC	Plant	1.40	0.2415	0.08	0.7778	11.62	**0.0011**
	Aphid	8.82	**0.0041**	0.17	0.6847	4.02	**0.0489**
Phloem							
n_E1	Plant	0.07	0.7936	164.95	**0.0001**	10.91	**0.0015**
	Aphid	10.15	**0.0022**	77.37	**0.0001**	0.01	0.9320
s_E1	Plant	0.62	0.4349	7.27	**0.0088**	13.75	**0.0004**
	Aphid	2.56	0.1146	16.36	**0.0001**	7.79	**0.0068**
d_E1followedby1sE2	Plant	0.38	0.5423	301.01	**0.0001**	8.34	**0.0052**
	Aphid	8.05	**0.0060**	241.71	**0.0001**	3.69	0.0591
%_E1/E12	Plant	0.16	0.6866	9.08	**0.0036**	13.25	**0.0005**
	Aphid	2.49	0.1196	11.83	**0.0010**	9.55	**0.0029**
%probtimeinE1	Plant	0.01	0.9199	10.63	**0.0017**	10.96	**0.0015**
	Aphid	1.32	0.2548	21.41	**0.0001**	3.01	0.0871
s_E2	Plant	0.46	0.4986	1.39	0.2427	4.02	**0.0490**
	Aphid	0.99	0.3241	0.32	0.5713	0.31	0.5788
s_longestE2	Plant	0.31	0.5793	13.35	**0.0005**	10.73	**0.0017**
	Aphid	1.39	0.2426	21.19	**0.0001**	4.93	**0.0298**
E2index	Plant	0.15	0.7038	11.04	**0.0014**	4.54	**0.0366**
	Aphid	1.62	0.2070	16.77	**0.0001**	0.43	0.5166
%sE2/E2	Plant	5.65	**0.0203**	91.89	**0.0001**	9.72	**0.0027**
	Aphid	15.36	**0.0002**	33.44	**0.0001**	1.69	0.1974
%probtimeinE2	Plant	0.36	0.5532	0.46	0.5000	10.24	**0.0021**
	Aphid	2.67	0.1068	1.04	0.3116	4.95	**0.0294**

^1^ Plant means comparison of infected plants and mock-inoculated plants; Aphid means comparison of viruliferous and non-viruliferous aphids.

## Data Availability

The associated data produced for this study can be cited: Zhong-Ping Hao. 2022. Turnip mosaic virus infection differentially modifies cabbage aphid probing behavior between spring and winter oilseed rape (*Brassica napus*) cultivars; Mendeley Data; V1; doi:10.17632/b568sjxtnn.1.
